# Planning a national-level data collection protocol to measure outcomes for the Colorectal Cancer Control Program

**DOI:** 10.21633/jgpha.6.2s16

**Published:** 2016

**Authors:** Anamika Satsangi, Amy DeGroff

**Affiliations:** Division of Cancer Prevention and Control, Centers for Disease Control and Prevention, Atlanta, GA

**Keywords:** colorectal cancer screening, evaluation, outcome

## Abstract

**Background:**

The Colorectal Cancer Control Program (CRCCP) of the Centers for Disease Control and Prevention (CDC) funded 30 grantees to partner with health systems with the goal of increasing screening for colorectal cancer (CRC).

**Methods:**

Evaluators applied CDC’s Framework for Program Evaluation to design a national level outcome evaluation for measuring changes in CRC screening rates in partner health systems.

**Results:**

The resulting evaluation design involves the collection and reporting of clinic-level CRC screening rates supplemented by various tools to support the reporting of high quality, reliable data.

**Conclusions:**

The CRCCP evaluation represents a strong design to measure the primary outcome of interest, CRC screening rates, and public health practitioners can benefit from lessons learned about stakeholder involvement, data quality, and the role of evaluators in data dissemination.

## INTRODUCTION

**Figure 1:**
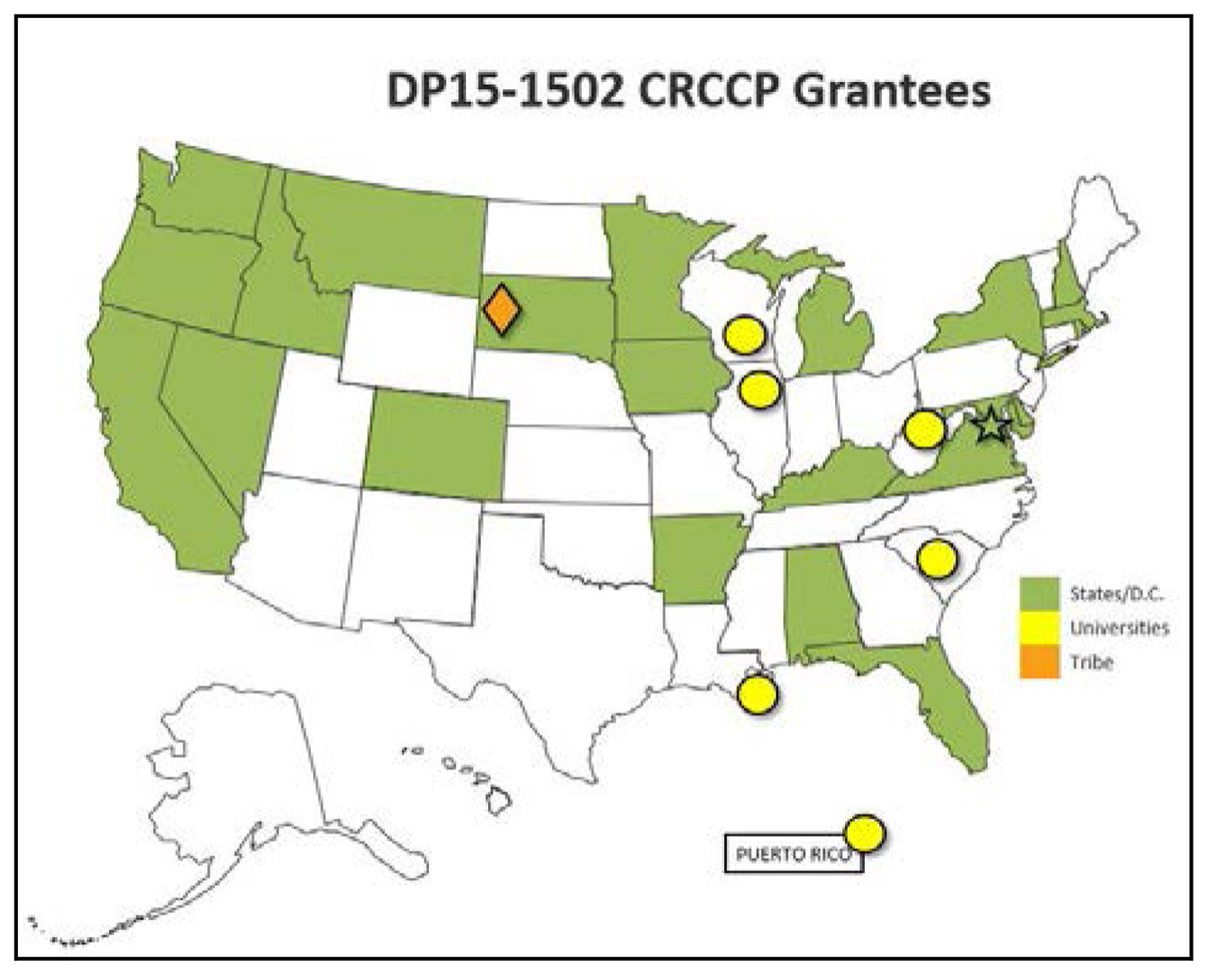
Map of CRCCP grantees

Colorectal cancer (CRC) is a leading cause of cancer-related death in the U.S. (U.S. Cancer Statistics Working Group, 2016). Although screening reduces CRC incidence and mortality (Whitlock, Lin, Liles, Beil, & Fu, 2008), screening rates remain low (CDC, 2014). To increase screening rates, the Centers for Disease Control and Prevention (CDC) funded the Colorectal Cancer Control Program (CRCCP) in 2015 for five years (CDC, 2016). Thirty grantees partnered with healthcare systems to implement evidence-based interventions (EBIs) such as provider and client reminders recommended in the Community Guide (Community Preventive Services Task Force, 2016) (Figure 1). This report describes an outcome evaluation designed to assess changes in screening rates in partner health systems.

## METHODS

CDC evaluators applied the Framework for Program Evaluation to design a national-level CRCCP evaluation (Figure 1) (CDC, 1999). The framework includes (1) engaging stakeholders, (2) describing the program, (3) focusing the evaluation design, (4) gathering credible evidence, (5) justifying conclusions, and (6) ensuring use and sharing lessons learned.

Stakeholders, including CDC program consultants, leaders, CRCCP grantees, and healthcare experts, helped to define the purpose of the evaluation (i.e., program improvement, accountability) and provided guidance throughout the evaluation planning process. Evaluators created a program logic model (Appendix) describing CRCCP activities and outcomes that helped focus the design. Using the logic model, process and outcome evaluation questions were drafted and vetted with stakeholders. The primary outcome evaluation question was, “Do CRC screening rates increase in CRCCP partner health systems?” To examine this question, evaluators determined that screening rates would be assessed annually over the five-year program period. In consultation with stakeholders, evaluators learned that many grantees planned to work with subsets of primary care clinics within given health systems rather than all clinics in a given system. Therefore, clinic-level screening rate data (vs. health system-level) were needed.

Evaluators developed a data dictionary detailing the variables to be reported to CDC by grantees along with data collection tools and guidance documents. Variable selection was informed by the evaluation purpose and questions. With five grantees, materials were pilot-tested to assess clarity, feasibility, and, for tools, functionality. Based on pilot testing, needed changes were incorporated. Evaluators also solicited advice from several national healthcare experts. Strategies to strengthen data quality were incorporated into the evaluation design. Finally, evaluators developed an analysis plan and selected dissemination strategies to ensure feedback of evaluation results.

## RESULTS

Grantees reported baseline clinic-level data, including screening rates, for all clinics participating in the CRCCP. Given the longitudinal evaluation design, grantees also reported screening rates annually for each clinic through the end of the cooperative agreement. The data dictionary was comprised of 110 variables, including health system and clinic identification codes used to link records over time. Other variables captured descriptive data (e.g., health system name, clinic name, number of patients) and longitudinal data (e.g., screening rate, EBI implementation).

Grantees calculated screening rates by medical chart review and/or electronic health record data. CDC evaluators developed a guidance document for grantees to support the consistent and accurate measurement of screening rates (CDC, 2016). For each clinic, grantees defined the 12-month measurement period (e.g., calendar year) and chose one of four screening rate measures recommended by CDC (e.g., National Committee for Quality Assurance, Health Resources and Services Administration). The guidance document also offered strategies to validate the screening rate.

Excel-based data collection forms were created, and grantees used them to gather baseline and annual data (Appendix 2). To improve data quality, these forms incorporated validation features (e.g., specified ranges, drop-down boxes). To report clinic data, grantees used a web-based data reporting system, Clinic Baseline and Annual Reporting System (CBARS), which incorporates similar data field edit checks to strengthen data quality. To support grantees in their data collection and reporting, evaluators conducted webinars, provided individual technical assistance, and maintained a document of frequently asked questions.

Baseline data for clinics recruited in program year 1 were analyzed by CDC, and reports were developed for stakeholders. Future dissemination efforts will use data visualization software that allows grantees to examine their own data.

## DISCUSSION/CONCLUSIONS

Representing the integration of public health and primary care, the CRCCP offers an opportunity to increase CRC screening. Using CDC’s Framework for Evaluation, a strong evaluation has been designed to assess the CRCCP’s primary outcome of interest, CRC screening rates, using medical record data.

Several lessons can be derived from this experience. First, conducting high quality, systematic outcome evaluations of Federal programs such as the CRCCP is difficult when many grantees and potentially hundreds of implementation sites are involved. Such scenarios inherently involve data access and quality challenges (DeGroff, Schooley, Chapel, & Poister, 2010). However, broad stakeholder involvement ensured that CDC crafted a meaningful outcome evaluation question, identified a feasible data collection strategy that was not overly burdensome, and selected data variables accessible to all participating health system clinics. Second, CDC evaluators integrated various strategies to ensure data quality and strengthen reliability, including developing a data dictionary with standardized variable definitions, developing guidance on how to measure screening rates, providing data collection forms and a web-based reporting system with built-in validation features, and delivering various types of technical support. Finally, evaluators have a critical role to play in data use such as facilitating interpretation. For the CRCCP, data feedback mechanisms are in place, with more sophisticated dissemination efforts being planned using data visualization software. Timely dissemination of data to grantees in a digestible fashion enables meaningful data feedback and use, and reinforces the importance of grantees reporting high-quality data.

Evaluation of public health programs is essential to ensure accountability to stakeholders, including funders, and to improve programs. Good evaluation planning is foundational to realizing these aims. Public health practitioners and evaluators can apply CDC’s Framework for Program Evaluation and the lessons identified here to support their own evaluation planning.

## Figures and Tables

**Figure 2:**
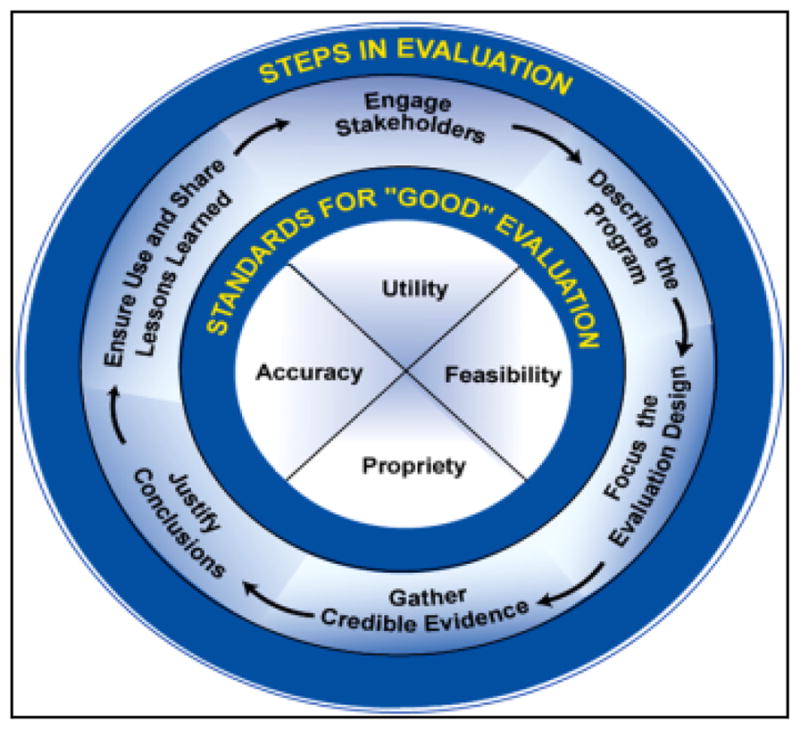
CDC Framework for Program Evaluation
